# Effects of Nutrition Intensification on the Secondary Ovary Development and Oviposition of Redclaw Crayfish

**DOI:** 10.1155/2024/8347388

**Published:** 2024-08-24

**Authors:** Cheng Shun, Chi Mei-li, Zheng Jian-bo, Jiang Wen-ping, Liu Shi-li, Hang Xiao-ying, Peng Miao, Li Fei, Wang Dan-li

**Affiliations:** ^1^ Zhejiang Institute of Freshwater Fisheries Agriculture Ministry Key Laboratory of Healthy Freshwater Aquaculture/Key Laboratory of Freshwater Aquatic Animal Genetic and Breeding of Zhejiang Province, Huzhou, Zhejiang 313001, China; ^2^ School of Marine Sciences Ningbo University, Ningbo, Zhejiang 315211, China

## Abstract

To further explore the impact of nutrient fortification on the ovarian secondary development of redclaw crayfish, four groups were set up: group ①, no feeding; group ②, formulated feeding; groups ③ and ④, formulated feed + nutrient bait. Results showed (1) the proportions of egg-bearing shrimp in groups ③ and ④. 2) The weight growth rate of group ① was the lowest, the maturation coefficient of unripe shrimp in group ④ was the highest, and the egg-holding rates in groups ③ and ④ were significantly higher than those of group ①. The hatching and survival rates of yellow eggs in group ④ were significantly higher than those in group ②. The hatching and survival rates of red eggs in groups ③ and ④ were significantly higher than those in group ①. (3) The weight gain rate of juveniles in group ④ was significantly higher than those of group ①. (4) Genes and proteins related to ovarian development were screened. In summary, group ④ had a higher proportion of egg-holding shrimp, faster ovarian development, and a higher maturity coefficient. The hatching and survival rates of eggs, and the survival and weight gain rates of the offspring were also high.

## 1. Introduction

Redclaw crayfish (*Cherax quadricarinatus*) is an economically important freshwater crayfish with considerable market potential, and its aquacultural development in China has recently been growing rapidly [1, 2]. Currently, breeding methods include indoor cement pond breeding and outdoor soil pond breeding. In nontropical areas, indoor cement pond breeding is preferred because this method has a higher and more stable emergence, a controllable environment, and a high degree of intensification, making it more suitable for a tropical species, such as the redclaw crayfish [3, 4]. However, because of the low output of seedling capacity and the laggard large-scale seedling breeding technology, the supply of shrimp seedlings is reduced; therefore, conducting targeted research to solve this bottleneck problem is urgent.

Since the introduction of shrimp in the 1990s, China has developed effective artificial breeding systems through exploration. In the October of each year, the parent crayfish entered the greenhouse and are bred for overwintering until the following year for mating. The crayfish are then cultivated until the first batch of seedlings are produced, and postpartum crayfish are mated again [3]. Each female mates and spawns more than three times during its breeding cycle [5]. To adapt to the breeding time, it starts to mate in February, and the first batch of crayfish with eggs is caught 1 month later. This batch of crayfish become postpartum crayfish after 15–40 days, and a second mating is carried out. Because of the large emergence time span, large differences in specifications, and low water temperatures in the outer pond, the juveniles produced by the first batch of crayfish need to be cultured in a warm shed. After another month, a second batch of crayfish with eggs are caught. These become postpartum crayfish after 20–30 days (the juveniles produced by this batch of crayfish are regular in specifications and can be directly placed into the pond for cultivation). Juveniles produced by the third and fourth batches of crayfish with eggs, owing to the late emergence time, small size, and decline in quality [6], result in insufficient demand from farmers. Therefore, the key to the success of the entire reproduction process mainly depends on the first two batches of crayfish with eggs. However, the first batch of crayfish with eggs come from parent shrimp after a long winter of cultivation, which is affected by many factors. Unlike the first batch, the second batch of crayfish with eggs originates from postpartum crayfish mating. We believe that nutritional enhancement (adequate supply of animal and plant food) in this relatively short period could have a significant impact on the improvement of ovary development and oviposition.

Ovary development to maturity is a process involving massive nutrient accumulation, and the demand for nutrients during the reproductive period is much higher than that during the growth period [7]. During the mature period of ovarian development, the accumulation of sufficient nutrients is the basis for ensuring the normal development of eggs, as well as material guarantee for improving the egg-holding capacity, hatchability, and survival rates of juveniles [8, 9]. However, to maintain basic life activities, crayfish might reabsorb the nutrients in the gonads and convert them into the energy needed for life activities, thereby blocking gonad development [10]. Harrison [11] stated that adequate nutrition and energy in the parent diet are necessary for the maturation of gonads and affect the composition of the ovaries and the quality of eggs. The main nutrients stored in the body of parent crayfish before gonadal development are proteins, amino acids, and lipids (mainly phospholipids), which are transferred to the gonads for use during gonadal development [12, 13]. Proteins are mainly used for gonadal development, and lipids mainly meet energy requirements [14].

Harlıoğlu and Farhadi [15] reported that the diet of *Pacifastacus leniusculus* should contain sufficient protein and essential amino acids to meet its growth and yolk development needs. Li et al. [16] showed that diets with high protein content could significantly improve the oviposition of redclaw crayfish. In addition to proteins, gonadal development is mainly affected by lipids, carotenoids, and other substances in food [17]. Lipids are the most important energy sources that are oxidised and decomposed for energy consumption during starvation or reproduction [18]. Rodríguez-González et al. [19] and Li et al. [16] concluded that the lipid demand of the gonad is partly fulfilled by food because the lipid reserves of the liver and pancreas cannot meet the demands of the ovary. Carotenoids also play a crucial role in gonadal development in redclaw crayfish [19]. Liñán-Cabello et al. [20] suggested that carotenoids (*β*-carotenoids and astaxanthin) are related to oocyte development, and carotenoids should be supplemented in the diet during breeding. Feeding a variety of mixed diets to the parent crayfish during the breeding period would accelerate the accumulation of nutrients in the parent crayfish, which is conducive to egg-holding by females, significantly improves the gonadal maturation and egg-laying ability of the parent crayfish, and shortens the time required for egg laying [21]. Zheng et al. [22] found that the quality and number of eggs held improved when snails and fish were supplemented daily. Zhang et al. [23] conducted a study on the effects of diet on the gonadal development of parents of *Procambarus clarkii* and found that the gonad development of females fed animal food was significantly better than that of females fed mixed pellet feed. Gendron et al. [24] showed that during the breeding period of *Homarus americanus*, strengthening the feeding of animal food was necessary to provide adequate nutrition to shrimp. Yu et al. [25] showed that the mixed feeding of plant-and animal-based diets was more conducive to promoting ovarian development in a study on the ovarian development of *P. clarkii*. Some bioactive factors, such as methylfarnesol in the body of the sand silkworm, could regulate the physiological metabolism level of the parent and significantly promote nutrient accumulation in the parent shrimp [26]. The research showed that the mating rate, spawning amount, and continuous spawning rate of parent shrimp improved to a greater extent when provided with sand silkworms, oysters, and squid, or a mixture of all three, than when only oysters were provided. Simultaneously, the time required for the parent shrimp to spawn again was shortened [27]. Therefore, an adequate supply of animal food rich in proteins, lipids, and plant food rich in carotenoids is the nutritional basis for ensuring gonadal maturity and egg quality of parent shrimp.

However, the application of histological methods, omics analysis, and other technical methods provide a feasible means of exploring the mechanisms of ovarian and embryonic development. Through histological analysis, Lu et al. [28] found that most of the ovaries of *P. clarkii* fed with diets containing 35.77%–41.12% protein developed to the fifth stage, and the frequency of mature oocytes in this stage was higher than that of other groups. Liu et al. [29] found that crustacean hyperglycemic family hormones, ecdysone, juvenile hormone, insulin-PI3KAkt, epidermal growth factor receptor, and transforming growth factor-*β* signal pathways played a crucial role in regulating ovarian development. Feng et al. [30] used a label-free quantitative method and conducted a comparative proteomic analysis on the ovary and hepatopancreas of *Eriocheir sinensis* at three key stages of ovarian development (oogonia proliferation, endogenous vitellogenesis, and exogenous vitellogenesis). A total of 2,224 proteins were identified, and some key proteins related to ovarian development and nutritional metabolism were differentially expressed. These findings provide new insights into the regulatory pathways and physiological processes involved in ovarian development in crustaceans. Therefore, strengthening nutrition during the secondary development of the ovary, ensuring an adequate supply of animal and plant food, and conducting in-depth analyses using histological methods, omics analysis, and other technical means is of great significance to improve large-scale seedling technology. The objective of this study was to increase the egg-holding rate, egg-holding amount, egg quality, emergence, and body constitution of juveniles by adding a certain proportion of hairtail, snail meat, sand silkworm, and carrot to their diet. With the secondary development of the ovary as the starting point, we screened the appropriate diet formula and nutritional fortification methods and explored the effects of nutritional fortification on the secondary egg-holding effect, egg quality, histological, physiological, and biochemical indicators of secondary ovarian development, as well as the quality of offspring. We combined transcriptome and proteomic analyses to comprehensively explore the effects of nutritional fortification on the secondary development of the ovary.

The success or failure of breeding mainly depends on the hatching and reproduction of the first two batches of egg-laying shrimp, wherein the first batch of egg-laying shrimp underwent long wintering and species conservation and was influenced by many factors. The discussion of the second batch of egg-laying shrimp was more targeted, and research focusing on this period has rarely been reported. At the same time, an adequate supply of animal food rich in proteins, lipids, and plant food rich in carotenoids as the nutritional basis has been proven to ensure the gonadal maturity of parent shrimp and the quality of fertilised eggs. Therefore, the results of this study have important practical significance and large application prospects that could be applied to production practices, academic value, and reference significance for freshwater crayfish and other batch spawning species.

## 2. Materials and Methods

### 2.1. Cultivation Test

The experiments were conducted at the Zhejiang Institute of Freshwater Fisheries in Huzhou, China. The redclaw crayfish were obtained from the Balidian breeding base of the Zhejiang Institute of Freshwater Fisheries (Huzhou, Zhejiang Province). The redclaw crayfish were anesthetized for 10 min in water with a mass concentration of 400 mg/L of eugenol. The anesthesia time referred to the time required for the crayfish to lost all their walking ability and maintain balance after entering the anesthesia water body and had no response to the stimulation of the glass rod.

Four groups were tested:

  Group ①: no feeding group
  Group ②: formulated feed group fed twice a day in the morning and evening. The amount of feed in the morning was 2% of the total weight of crayfish, and the amount of feed in the evening was 4% of the total weight of crayfish.
  Group ③: nutrition intensification on every other day, fed formulated feed in the morning, which was 2% of the total weight of crayfish. The feeding sequence in the evening was as follows: formulated feed on the first day, hairtail on the second day, formulated feed on the third day, snail meat on the fourth day, formulated feed on the fifth day, sand silkworm on the sixth day, formulated feed on the seventh day, frozen artemia on the eighth day, formulated feed on the ninth day, formulated feed + carrots on the tenth day, repeated again after the tenth day. The feeding amount of formulated feed in the evening was 4% of the total weight of crayfish, and the feeding amount of other feeds was based on when the crayfish had consumed the feed.
  Group ④: daily nutrition intensification, fed formulated feed in the morning, which was 2% of the total weight of crayfish. The feeding sequence in the evening was as follows: formulated feed + carrots on the first day, hairtail on the second day, snail meat on the third day, sand silkworm on the fourth day, and frozen Artemia on the fifth day, repeated again after the fifth day. The feeding amount of formulated feed in the evening was 4% of the total weight of crayfish, and the feeding amount of other feeds was based when crayfish had consumed the feed.

**Figure 1:**
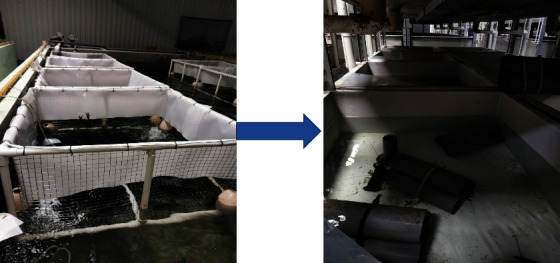
Crayfish breeding in floating tank and mating in circulating water tank.

The first batch of crayfish egg-holding that only held eggs was selected (the egg-holding condition was consistent, and the crayfish were faint yellow when they developed to the cleavage stage); ten tails in each group were placed into the buoyancy tank (in the same cement pool) and fed according to the four groups. The feed was fed into the tray of the buoyancy tank, and black plastic pipes were placed in each buoyancy tank as a shelter to ensure that the dissolved oxygen, temperature, and water quality of the breeding environment were in good condition for postpartum crayfish. Postpartum crayfish were removed and placed in a circulating water tank. Ten postpartum females and three males were placed in each tank for mating (weight, health status, nutritional level, etc., of the males were essentially the same). They were fed according to the four groups (Figure 1). During this period, the same concealed objects (nine plastic tubes per tank) were used to ensure that the dissolved oxygen, temperature, and water quality of the breeding environment were in good and consistent condition. Contaminants were absorbed daily. After 30 days, the females in each group were tested and sampled.

### 2.2. Histological Observation of Ovarian Secondary Development

Thirty days after mating, the ovaries of the four groups of females were selected for histological observation. The samples were stored in Bonn's solution. The samples were routinely embedded in paraffin and continuously crosscut with a slice thickness of 6 *μ*m. They were then stained with haematoxylin and eosin, observed under a microscope, and photographed. The development of crayfish ovaries without eggs and the proportions of different ovarian stages in each group were analysed. Finally, the ovaries of each group were divided into three stages: (stage A (recovery stage), stage B (maturity stage), and stage C (development stage)). The proportions of ovaries in each development stage of each group were counted, and the characteristics of ovaries in each stage were analysed.

### 2.3. Detection of Secondary Egg-Holding and Egg-Hatching Effect

#### 2.3.1. Female Crayfish Indicators

All indices of females in each group were tested, including body weight growth rate ((bodyweight−initial body weight)/initial bodyweight × 100%), hepatopancreas somatic indices (hepatopancreas weight/bodyweight × 100%), maturity coefficient (ovary weight/bodyweight × 100%), and maturity coefficient of unripe shrimp (ovary weight/bodyweight × 100%). The growth and gonadal development of female shrimps under different nutrient reserves were analysed.

#### 2.3.2. Egg-Holding Indicators

The egg-holding rate (number of egg-holding shrimp/number of female shrimp × 100%), absolute fecundity (average number of eggs per egg-holding shrimp), relative fecundity (absolute fecundity/body mass of female shrimp), yellow egg ratio (number of five pairs of appendage and previous eggs/total eggs × 100%), red egg ratio (number of seven pairs of appendage and subsequent eggs/total eggs × 100%), bad (dead, mouldy, and deformed) egg ratio (number of bad eggs/total number of eggs × 100%), and the egg-holding status of female shrimp under different nutrient reserves were analysed.

#### 2.3.3. Hatching Index of Eggs

The incubation system consisted of a recirculating mechanical pulling device and a homemade incubator box [31]. Eggs from each group were placed in an incubator box. The water could be effectively filtered, purified, and controlled at a temperature of 28°C–29°C. After emergence, the hatching rate (number of stage 1 juveniles/number of eggs × 100%) and survival rate (number of stage 3 juveniles/number of eggs × 100%) of the yellow and red eggs in each group were determined.

#### 2.3.4. Antioxidant Index Test

Five pairs of appendage eggs of each group were put into liquid nitrogen, and then the liquid nitrogen preservation samples were stored at −80°C. Samples were prepared by adding 9 mL normal saline to 1 g of the test sample. After grinding, the samples were transferred to 2 mL centrifuge tubes and centrifugation was performed at 3,000 rpm for 10 min at 4°C. Thereafter, the supernatant was separated and stored in a refrigerator at 4°C until subsequent assays. The concentrations of antioxidant indices (superoxide dismutase (SOD) and malondialdehyde (MDA)) were determined. The enzyme activity was determined using a commercial kit (Nanjing Jiancheng Bioengineering Institute, Nanjing, China).

### 2.4. Detection of Survival Rate and Growth Rate of Offspring in Each Group

Three hundred juveniles of similar specifications and healthy constitutions were taken from each group, and 300 juveniles from each group were randomly divided into 100 juvenile groups, which were placed in a circulating water tank for cultivation (3 parallel for each group, a total of 12 tanks). During this period, the same shelter (one mesh, one gusset shrimp house, and one black plastic tube) was used to ensure that the dissolved oxygen, temperature, and water quality of the cultivation environment were good. Consistent feed was provided once in the evening (the daily feeding amount was 10% of the total weight of juveniles) and dirt was absorbed every day. The feed amounts in each group were the same. After 30 days, the survival rate (number of juveniles/100 × 100%), weight gain rate ((final weight of juveniles−initial weight of juveniles)/initial weight of juveniles × 100%), and specific growth rate (SGR) ((Ln final weight of juveniles−Ln initial weight of juveniles)/cultivation time × 100%) of each group were counted.

### 2.5. Transcriptome Analysis of Ovary

Based on the results of histological observations, the ovaries at stages A (recovery stage), B (maturity stage), and C (development stage) were used for transcriptome analysis, and each development stage was performed in triplicate.

#### 2.5.1. RNA Extraction from Ovarian Tissue

RNA was extracted from ovarian tissues at different developmental stages using TRIzol® Reagent agent kit. The extracted RNA met the OD260/OD280 of 1.8–2.2, and the total RNA ≥ 5 *μ*g, concentration ≥ 200 ng/*μ*L, RIN ≥ 8. The library is constructed in the next step.

#### 2.5.2. Library Construction and Sequencing

A TruSeq RNA Sample Preparation Kit (Illumina) was used to construct an ovarian cDNA library. Five microgram total RNA, enriched mRNA with magnetic beads with Oligo dT, and fragmentation buffer was added. The obtained mRNA was randomly broken into small fragments of about 200 bp. Double-stranded cDNA was synthesised by the reverse transcription of small mRNA fragments using random primers. End Repair Mix was added to level the end, add A at the 3'end, and connect the connector. PCR amplification (15 cycles) was performed after the purification of the linkage product. The amplification product was used to recover ~200 bp of the target band on a 2% agar gel and a double-ended library for sequencing was obtained. The library was quantified using TBS380 Picogren, and bridge PCR amplification was performed using a cluster formation kit (TruSeq PE Cluster Kit v3-cBot-HS) from Illumina. After the cluster formation reaction, the high-throughput sequencing platform Illumina HiSeqTM 4000 was used for 2 × 151 bp sequencing. The obtained sequencing data were subjected to quality control.

#### 2.5.3. Assembly of Transcriptome De Novo

The original image data obtained by Illumina sequencing were converted into sequence data through Base Calling, and the raw data were filtered to remove the reads containing the sequencing connector, low-quality reads (alkali base with a quality value ≤10 accounting for more than 20% of the whole read), reads with an N rate of ≥10%, and short sequences in order to obtain high-quality sequencing data (clean reads). Trinity software was used to assemble the clean reads using default settings. First, the reads were spliced into long contigs, redundant contigs were removed, and the contigs were grouped into those that could no longer be extended at either end (unigenes).

#### 2.5.4. Transcriptome Notes

Before annotation, the open reading frame (ORF) prediction process provided by Trinity software was used to predict the genes of all assembled transcript sequences. The assembled transcripts were annotated in the Pfam database using HMMER3 program. The expected value <1e−5 was set and BlastX software was used to compare all nucleotide sequences obtained by splicing with the cluster of orthologous groups of proteins (COG), Gene Ontology (GO), Kyoto Encyclopaedia of Genes and Genomes (KEGG), clusters of orthologous groups for eukaryotic complete genomes (KOG), Pfam protein families (Pfam), Swiss-prot, evolutionary genealogy of genes (eggNOG), and nonredundant protein (NR) databases to obtain the corresponding annotation information.

#### 2.5.5. Analysis of Differential Gene Expression

Bowtie software was used to compare clean reads to the transcriptome sequence, and RSEM software was used to perform statistical analysis of the expression of the bowtie alignment results. The RPKM values (reads/fragments per kilobase of the xon model per million mapped reads) were used to measure gene expression. EdgeR software was used for gene expression difference analysis. After comparison of different samples, if the false discovery rate (FDR) value ≤0.05 and the logarithm of the differential multiple of gene expression amount with two as the base | log2FC (Sample2/Sample1) |≥1, the gene was a significantly differentially expressed gene. The transcriptome sequencing library at three stages of ovarian development was compared and analysed in pairs (C vs. A means that the case was C and the control was A, B vs. A means that the case was B and the control was A, B vs. C means that the case was B and the control was C).

#### 2.5.6. Gene Ontology (GO) Classification and Pathway Enrichment Analysis

After identifying the differentially expressed genes (DEGs), GO function and KEGG Pathway significance were analysed. Blast2go software was used to classify and annotate the biological processes, cell components, and molecular functions of the unigenes in the GO database. The Kyoto Encyclopaedia of Genes (KEGG) was used to obtain annotation information of the metabolic pathways of the unigenes.

#### 2.5.7. Real-Time PCR Validation

Through total RNA extraction, RNA concentration determination, cDNA synthesis, and real-time PCR, the relative expression levels between samples were obtained and displayed in a bar chart. The sample cDNA obtained by reverse transcription was diluted four times, the internal reference gene 18 s RNA was selected, and the SYBR reagent method was used to analyse the expression of the obtained samples. Fluorescent 96-well plates were placed in an ABI 7500 real-time PCR apparatus for amplification. The fluorescence quantitative PCR reaction procedure is as follows: 95°C, 30 s; 95°C, 5 s, 60°C, 60 s, 40 cycles. At 95°C for 5 s, 60°C for 1 min, the dissolution curve was detected by 2−*ΔΔ*CT.

### 2.6. Proteomic Analysis of Ovary

Based on the histological results, the ovaries at A (recovery stage), B (maturity stage), and C (development stage) were used for proteomic analysis, and the developmental stages were paralleled.

#### 2.6.1. Marker Quantitative Proteomics

Ovarian tissues at different developmental stages were identified by histological observation (A, recovery stage; B, maturity stage; and C, development stage). Part of the total protein in the sample was taken for protein concentration determination and Sodium dodecyl sulfate polyacrylamide gel electrophoresis (SDS-PAGE) detection, and the other part was taken for trypsin digestion and labelling. The same amount of labelled samples was used for chromatographic separation after mixing, and LC-MS/MS analysis and data analysis were conducted on the samples.

#### 2.6.2. Bioinformation Analysis

Qualitative and quantitative data were collected, and expression levels and functional analyses were conducted after quality evaluation and preprocessing. Several common databases were used to annotate and analyse the identified proteins. GO, pathway, and interaction analyses were carried out for differentially expressed proteins. Simultaneously, correlation analysis, expression pattern clustering heat map, and Venn analysis were conducted on the data of the comparison group.

#### 2.6.3. Protein Function Analysis

For the identified proteins, the annotation information was extracted based on Uniprot, KEGG, GO, KOG/COG and other databases, and the protein functions were mined. The differentially expressed proteins were enriched and analysed using GO and KEGG, and their functions were described. To screen for the key proteins and pathways involved in the secondary development of the ovary, we analysed the nutritional accumulation and gonadal development of these key proteins.

#### 2.6.4. ELISA Validation

A double-antibody one-step sandwich enzyme-linked immunosorbent assay (ELISA) was used. Samples, standards, and horseradish peroxidase (HRP)-labelled detection antibodies were added to the pre-coated target antibody-coated wells, followed by incubation and thorough washing. The tetramethylbenzidine (TMB) substrate was used for colour development. TMB was converted to blue under the catalysis of peroxidase and finally to yellow under the action of an acid. The depth of colour was positively correlated with the protein in the sample. The absorbance was measured using an ELISA at a wavelength of 450 nm to calculate the sample concentration.

### 2.7. Statistical Analysis

All data are presented as mean ± standard deviation (SD). After arcsine transformation, the data obtained for each experiment were analysed by analysis of variance (ANOVA) using SPSS software (version 17.0; IBM, Armonk, NY, USA) to determine group differences. Tukey's multiple comparison post hoc test was performed when significant differences were detected. Statistical significance was set at *P* ≤ 0.05 for all analyses.

## 3. Results

### 3.1. Nutritional Fortification on Histology of Ovarian Secondary Development

**Figure 2:**
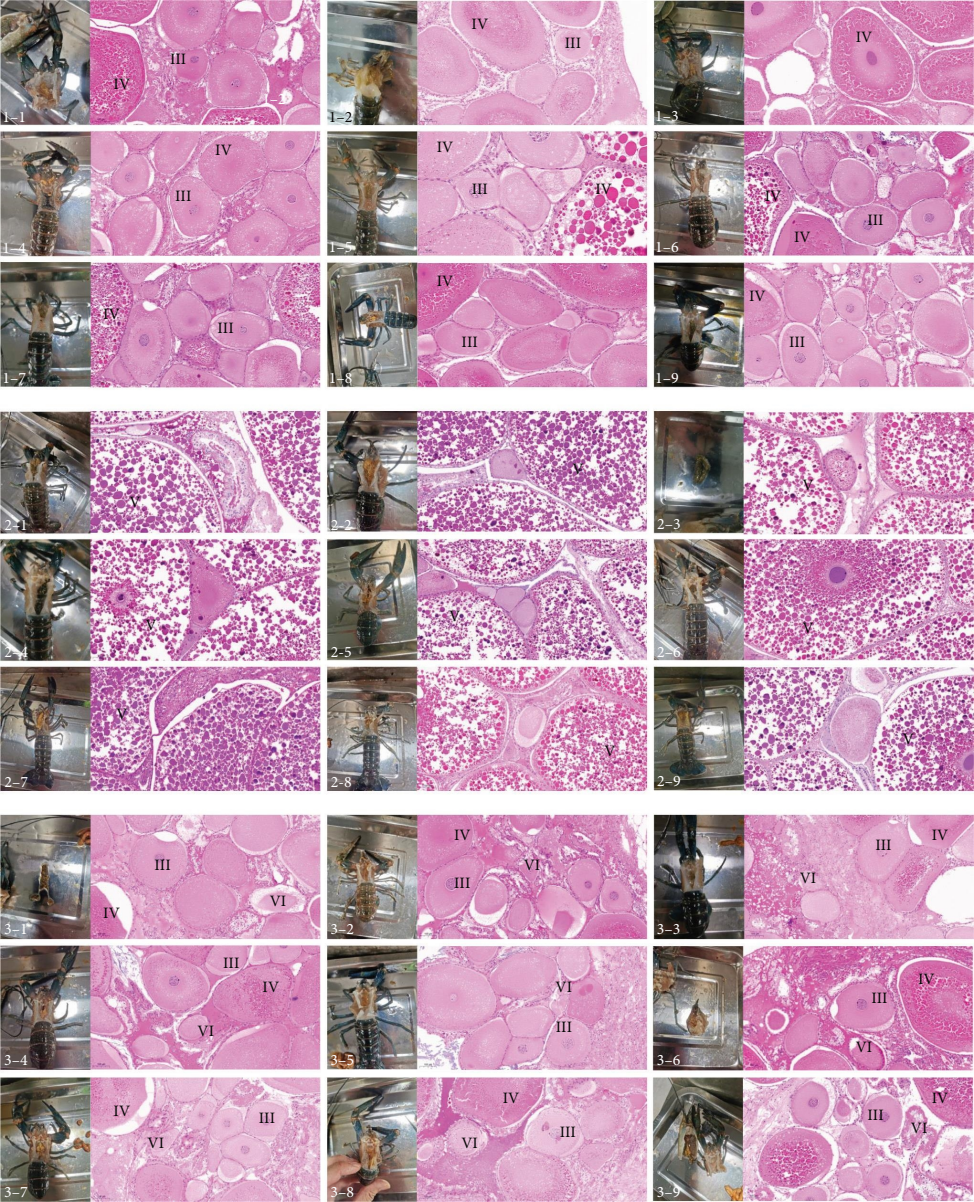
Ovaries in different stages. 1–1 to 1–9, ovaries in the developmental stage; 2–1 to 2–9, ovaries in the mature stage; 3–1 to 3–9, ovaries in the recovery stage (III, Phase 3 oocyte; IV, Phase 4 oocyte; V, Phase 5 oocyte, namely mature oocyte; VI, Phase 6 oocyte, namely degenerated oocyte).

The histological sections showed that stage C (developmental stage) ovaries in each group were dominated by oocytes in phases III and IV, and some ovaries were later found in oocytes in phase IV. In stage B (mature stage), the ovaries were phase-V oocytes. The ovaries in stage A (recovery stage) were dominated by oocytes in phases VI, III, and early phase IV, of which phase-VI oocytes were degraded, absorbed, and redeveloped (Figure 2).

Through the detection of ovaries and observation of histological sections, the ovaries in group ① were mainly in the developmental and maturity stages, and the ovaries in the recovery stage accounted for only 12.22%. The distribution of ovaries in group ② was uniform, and the proportions of ovaries in the developmental, mature, and recovery stages were similar. The ovaries in group ③ were mainly in the recovery stage, and the proportion of ovaries in the developmental stage was close to that in the mature stage. The ovaries in group ③ were mainly in the recovery stage, followed by those in the mature stage. Ovaries in the developing stage accounted for only 14.48% of the ovaries (Table 1). In other words, the proportion of egg-holding crayfish in groups ③ and ④ was high; therefore, the ovaries of these two groups were mainly in the recovery stage, and the proportion of mature stage ovaries in group ④ was significantly higher than that in group ③. The ovaries in group ④ developed rapidly. Ovarian development in group ② was very balanced, and the proportion of each stage was similar. The ovaries of group ① were affected by starvation; there were few egg-holding crayfish, and the proportion of ovaries in the recovery stage was low. Therefore, the ovaries develop slowly, particularly during the developmental and mature stages.

### 3.2. Nutrition Intensification on the Effect of Secondary Embracing, Hatching, and Antioxidant Index of Egg

#### 3.2.1. Female Crayfish Index

The results showed that the bodyweight growth rate of group ① was significantly lower than that of the other three groups (*P* < 0.05). The maturation coefficient of unripe shrimp of group ④ was significantly higher than that of the other three groups (*P* < 0.05), indicating that the ovary maturity of unripe shrimp of group ④ was high (Table 2).

#### 3.2.2. Egg-Holding Index

The results showed that the egg-holding rates in groups ③ and ④ were significantly higher than that in group ① (*P* < 0.05), and the bad eggs rate in group ④ was significantly lower than that in group ① (*P* < 0.05). This shows that nutrition enhancement could promote an increase in the egg-holding rate and reduce the proportion of dead, mouldy, and deformed eggs (Table 3).

#### 3.2.3. Hatching Index of Egg

The hatching and survival rates of yellow eggs in group ④ were significantly higher than those in group ② (*P* < 0.05). The hatching and survival rates of red eggs in groups ③ and ④ were significantly higher than those in group ① (*P* < 0.05). The survival rate of red eggs in group ④ was significantly higher than that in the other three groups (*P* < 0.05). Since group ① was red eggs, there was no data of group ① for comparison of yellow eggs (Table 4).

#### 3.2.4. Antioxidant Index of Egg

The SOD content in group ④ was significantly higher than that in groups ① and ② (*P* < 0.05), while the MDA content was significantly lower than that in groups ① and ② (*P* < 0.05). Nutritional enhancement might lead to the ability of eggs to resist oxidative stress and external stimuli (Table 5).

### 3.3. Effects of Nutrition Intensification on Survival Rate and Growth Rate of Offspring

The results showed that the survival rate of group ① was significantly lower than that of the other three groups (*P* < 0.05). The rate of weight gain in group ④ was significantly higher than that in group ① (*P* < 0.05). This suggests that nutrition promoted the survival and weight gain rates of the offspring (Table 6).

### 3.4. Transcriptome Analysis of Ovary

#### 3.4.1. Transcriptome Sequencing

**Figure 3:**
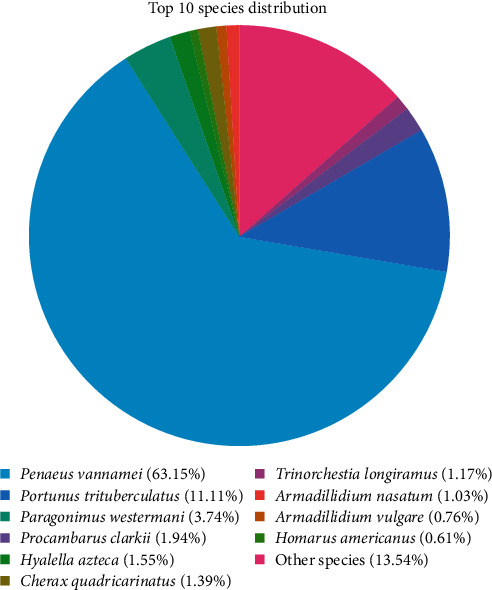
Species homology analysis.

1. Sequencing data statistics and assembly: Three separate sequencing libraries were established according to ovarian samples from three different stages of ovarian development. Through Illumina HiSeqTM 4000 high-throughput sequencing, the number of raw reads in the three stages of ovarian development were 135.8 M, 128.24 M, and 143.09 M, respectively. After filtering the original data, the number of clean reads in the three stages were 132.26 M, 125.02 M, and 140.18 M, respectively. Trinity software was used to assemble the transcriptome with de novo, and 42015 unigene was spliced with a total length of 48,384,068 bp, an average length of 1,151.59 bp, and average GC content of 42.18%. The statistical results of the length distribution of all unigenes showed that 10,235 unigenes were in the range of 301–400 bp, accounting for 24.36%; 6,883, accounting for 16.38%, were longer than 2,000 bp; and 5,953 were 401–500 bp in length, accounting for 14.17%.
2. Transcriptome notes: By comparison with the NR, COG/KOG, GO, Swiss Prot, eggNOG, KEGG, and Pfam databases, 78,860 unigenes were homologous to known genes. The numbers of homologous sequences in the NR, Swissprot, KEGG, KOG, eggNOG, GO, and Pfam databases were 17,428 (41.48%), 11,577 (27.55%), 5,013 (11.93%), 10,155 (24.17%), 12,601 (29.99%), 10,614 (25.26%), and 11,472 (27.30%), respectively. Species homology analysis showed that the redclaw crayfish unigenes in this study were homologous to the invertebrate genes in the NR database (Figure 3). Among the 1,742 unigenes compared with the NR database, the proportion of sequences homologous to *Penaeus vannamei* was the highest, reaching 63.15%. The second most abundant species was *Portunus trituberculatus*, reaching 11.11% (Figure 3).

#### 3.4.2. Differentially Expressed Genes (DEGs)

The transcriptome sequencing library at the three stages of ovarian development was compared and analysed in pairs. According to the logarithm value | log2FC (Sample2/Sample1) | ≥ 1 with the FDR ≤ 0.05 and the gene expression difference multiple based on 2 after comparing different samples, the gene differential expression at each stage was obtained. In total, 1,318 DEGs were detected, including 1,011 significantly upregulated and 307 significantly downregulated genes. Among these, the number of genes with significant differences in C vs. A expression was 110 (up 54, down 56), the number of genes with significant differences in B vs. A expression was 633 (up 502, down 131), and the number of genes with significant differences in B vs. C expression was 575 (up 455, down 120).

#### 3.4.3. GO Functional Classification Analysis of DEGs

A total of 3,564 DEGs were successfully annotated in the GO database. These were classified into 64 secondary categories based on their biological processes, cellular components, and molecular functions. In biological processes, the DEGs mainly focused on cellular processes and metabolic processes, cellular processes and biological regulation, and cellular processes and processes in the C vs. A, B vs. A, and B vs. C groups, respectively. In cellular components, the DEGs mainly focused on membranes and organelles, organelles and membranes, and membranes in the C vs. A, B vs. A, and B vs. C groups, respectively. In cellular components, the DEGs mainly focused on the membrane and organelle, organelle and membrane, membrane, and extracellular region in the C vs. A, B vs. A, and B vs. C groups, respectively. Regarding molecular functions, the DEGs mainly focused on binding and catalytic activity.

#### 3.4.4. Enrichment Analysis of KEGG Metabolic Pathway of DEGs

According to the KEGG metabolic pathway, the pathways with the highest enrichment levels in C vs. A were organismal systems-digestive system, metabolism-amino acid metabolism, metabolism-lipid metabolism, organismal systems-development and regeneration, cellular processes-cellular community-eukaryotes. The pathways with the highest enrichment levels in B vs. A were cellular processes-transport and catabolism, environmental information processing-signal transduction, organismal systems-endocrine system, human diseases-cancer: overview, organismal systems-digestive system. The pathways with the highest enrichment levels in B vs. C were cellular processes-transport and catabolism, human diseases-infectious disease: bacterial, human diseases-cancer: overview, organismal systems-endocrine system, environmental information processing-signal transduction.

#### 3.4.5. Screening of Genes Related to Ovarian Development

Different genes perform different biological functions in coordination with each other. Cathepsin L protein (CTSL) or vitellogenin receptor (VGR) was one of the major DEGs in C vs. A, *CTSL* was one of the major DEGs in B vs. A, and the CTSL or vitellogenin (VGR) gene was one of the major DEGs in B vs. C.

#### 3.4.6. The qRT-PCR Validation

**Figure 4:**
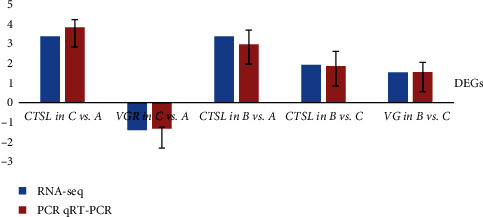
Comparison of seven differential expression genes by qRT-PCR and transcriptome analysis. CTSL, cathepsin L protein and VGR, vitellogenin receptor.

CTSL or VGR was one of the major DEGs in C vs. A, CTSL was one of the major DEGs in B vs. A, and CTSL or VG was one of the major DEGs in B vs. C. Next, we validated the results by qRT-PCR. The results showed that the detected gene expression trends were consistent with the transcriptome expression analysis results, proving that the RNA-seq results were reliable (Figure 4).

### 3.5. Proteomics Analysis of Ovary

#### 3.5.1. Differentially Expressed Proteins

A total of 1,585 differentially expressed proteins (DEPs) were identified. Among these, the number of proteins with significant differences in C vs. A expression was 68 (up 50, down 18), the number of genes with significant differences in B vs. A expression was 587 (up 512, down 75), and the number of genes with significant differences in B vs. C expression was 930 (up 626, down 304).

#### 3.5.2. Venn Analysis

Analysis of the characteristics and commonalities of differential proteins in each group using Venn analysis was convenient for the subsequent experimental design and selection of research directions. The results showed that there were 4 common differential proteins among the three groups: 19 common differential proteins in the B vs. A and C vs. A groups, 393 common differential proteins in the B vs. A and B vs. C groups, and 25 common differential proteins in the B vs. C and C vs. A groups.

#### 3.5.3. Protein Functional Analysis

Annotation information was extracted from databases such as Uniprot, KEGG, GO, KOG/COG for the identified proteins to explore their functions. After obtaining the DEPs, GO/KEGG enrichment analysis was performed on the DEPs to describe their functions.

1. GO enrichment analysis: In biological processes, the DEPs mainly focused on peptide cross-linking and innate immune response, cytoplasmic translation and microtubule-based processes, translation, and protein folding in the C vs. A, B vs. A, and B vs. C groups, respectively. In the cellular components, the DEPs mainly focused on the extracellular region and extracellular space, peroxisome and melanosome, cytosolic large ribosomal subunit, and ribosome in the C vs. A, B vs. A, and B vs. C groups, respectively. In molecular functions, the DEPs mainly focused on metal ion-binding and protein-glutamine gamma-glutamyltransferase activity, structural constituents of ribosome and cytoskeleton, structural constituent of ribosome, and unfolded protein binding in the C vs. A, B vs. A, B vs. C groups, respectively.
2. KEGG enrichment analysis: According to KEGG metabolic pathway analysis, the pathways with the highest enrichment levels in C vs. A were phagosomes, chemical carcinogenesis-DNA adducts, drug metabolism-cytochrome P450, metabolism of xenobiotics by cytochrome P450, and glutathione metabolism. The pathways with the highest enrichment levels in B vs. A were protein processing in the endoplasmic reticulum, longevity regulating pathway-worm, toxoplasmosis, glycolysis/gluconeogenesis, antigen processing, and presentation. Meanwhile, the pathways with the highest enrichment levels in B vs. C were ribosome, protein processing in the endoplasmic reticulum, endocytosis, glycolysis/gluconeogenesis, and necroptosis.

#### 3.5.4. Screening of Proteins Related to Ovarian Development

Different proteins perform different biological functions in coordination with each other. Serine proteinase inhibitor (SPI) was one of the major DEPs in C vs. A, VG, lipid storage droplet protein (LSDP), and SPI were the major DEPs in B vs. A and B vs. C.

#### 3.5.5. ELISA Validation

**Figure 5:**
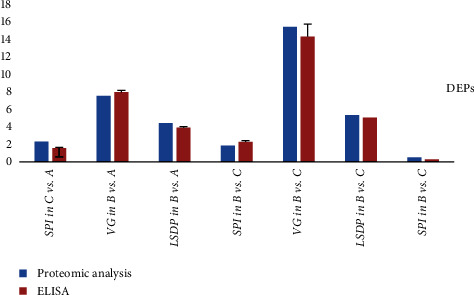
Comparison of seven differentially expressed proteins by proteomic analysis. ELISA, enzyme-linked immunosorbent assay; SPI, serine proteinase inhibitor; VG, vitellogenin.

SPI was one of the major DEPs in C vs. A, and VG, LSDP and SPI were the major DEPs in B vs. A and B vs. C. We conducted an ELISA validation. The results showed that the detected protein expression trends were consistent with the proteomic analysis results, proving that the proteomic analysis results were reliable (Figure 5).

## 4. Discussion

### 4.1. Histological Study and Genes Analysis of Secondary Ovarian Development by Nutritional Fortification

In this study, we found that the ovaries of groups ③ and ④ were mainly in the recovery stage, and the proportion of mature stage ovaries in group ④ was significantly higher than that in group ③. The proportion of each stage in group ② was similar. The ovaries of group ① were mainly in the developmental and mature stages. Studies have shown that accumulating sufficient nutrients during the development and maturation of the ovaries is the foundation for ensuring normal embryonic development and the nutritional needs of the seedlings, as well as the material guarantee for improving egg-holding capacity, hatching rate, and survival rate of seedlings [8, 9]. The main nutrients stored in the shrimp body before gonadal development are proteins, amino acids, and lipids, which are quickly transferred to the gonads for utilisation during gonadal development [12]. Therefore, group ④, for which food fortification was suitable, showed rapid ovarian development.

We compared ovaries in the developmental and recovery stages through deep exploration of related gene changes and found that the CTSL gene was significantly upregulated, whereas the VGR gene was significantly downregulated. After a period of recovery, the ovaries in the recovery stage resumed development, leading to ovaries in the development stage. This indicates that during the recovery and developmental stage, the expression of the CTSL gene significantly decreased, while that of the VGR gene significantly increased with the redevelopment of the ovaries. We compared ovaries in the maturity and recovery stages and found that the CTSL gene was significantly upregulated. After ovulation, mature ovaries rapidly atrophied and became ovaries in the recovery stage, indicating a significant decrease in the expression of the CTSL gene after ovulation in the mature ovaries. We compared ovaries in the mature and developmental stages and found that the CTSL gene and VG gene were significantly upregulated. Under conditions of sufficient nutrition and a suitable environment, the developing ovary would further develop until it became a mature ovary, indicating that the expression of the CTSL and VG genes significantly increases during the process of ovarian transformation from development to maturity.

Some studies have demonstrated a correlation between these genes and ovarian development. The CTSL gene belongs to the lysosomal papain family of cysteine proteases and is associated with the occurrence of yolk in shrimp and crab species, such as *Fenneropenaeus chinensis*, *Penaeus monodon*, *Exopalaemon carinicauda*, and *E. sinensis* [32, 33, 34]. In this study, we found that the expression of the CTSL gene experienced a decline from the recovery to the developmental stage of the ovary, increased from the developmental to the maturity stage, and declined from the maturity to the recovery stage, suggesting that CTSL might play a role in the process of sexual maturity of the ovary. The development of animal ovaries include three stages: the oogonial, pre-vitellogenesis, and vitellogenesis stages. Vitellogenesis is a key step in the accumulation of proteins, fats, and other nutrients [35]. VG is a precursor of vitellin. Exogenous VG binds to the VGR located in the cytoplasmic membrane of the oocytes to form a VG/VGR complex. After the complex is internalised into the cytoplasm, VG is processed into a mature yolk protein, and VGR is re-recruited to the cell membrane through tubules/follicles [35]. Ruan et al. [35] cloned the full-length cDNA sequence of VGR from *Litopenaeus vannamei* and characterised its gene structure and protein domain. We inferred that the VGR protein was located on the cell membrane of mature oocytes, whereas the accumulated VG protein was evenly distributed in the cytoplasm. The transfer of the VGR protein from the cytoplasm to the plasma membrane indicated that *L. vannamei* began to rapidly accumulate yolk, and siRNA silencing of the VGR transcript effectively inhibited *L. vannamei* ovarian development. In this study, we found that when the ovaries were in the recovery and development stages, the VGR gene expression significantly increased with the redevelopment of the ovaries, which might indicate that the ovaries rapidly accumulated yolk. In addition, another gene related to ovarian development, VG, was rapidly expressed afterwards. Ovarian development in crustaceans mainly involves the synthesis of VG through vitellogenesis, which in turn accumulates the corpus luteum and lipid droplets in oocytes [36]. In decapods, it mainly serves as a carrier protein for oocytes and provides essential nutrients for embryos and early larvae [37]. The expression levels of VG in *Penaeus japonicus* [38] and *Macrobrachium rosenbergii* [39] increase with an increase in the gonadal index. In *P. japonicus*, the expression of VG in the hepatopancreas decreases when the ovaries are fully mature [38]. Serrano-pinto et al. [40] found that the ovaries play an important role only in yolk protein production during certain stages of the yolk synthesis cycle. Okumura et al. [41] measured the expression levels of VG during ovarian development in *P. japonicus* and found that the GSI and VG mRNA levels were positively correlated with ovarian development. In this study, we found that the expression of VG genes significantly increased during the process of ovarian transition from development to maturity, which helped synthesise VG through vitellogenesis and accelerated the development and maturation of the ovaries.

### 4.2. Histological Study and Related Proteins Analysis of Secondary Ovarian Development under Different Levels of Nutritional Fortification

In this study, we found that group ④ had faster ovarian development. Deep exploration of related protein changes of ovaries in development recovery stages showed that SPI was significantly upregulated, indicating that the expression of SPI would significantly decrease after a period of recovery and redevelopment of the convalescent ovary. We compared ovaries in the maturity and recovery stages and found that VG, LSDP, and SPI were significantly upregulated, indicating that these would be significantly reduced as the mature ovary shrinks rapidly after oviposition and becomes convalescent. We compared ovaries in the mature and developmental stages and found that VG, LSDP, and SPI were also significantly upregulated, indicating that these would increase significantly during the development and maturation of the developing ovary. VTG is a precursor of the main component in the yolk of egg-laying animals, which reflects the maturity of female gonads and has significant implications for ovarian maturation and individual development [42]. In this study, we found that during the development and maturation of the developing ovary, the expression of VG significantly increased, causing the yolk to accumulate continuously until the ovary became mature. However, when the mature ovary became a recovering ovary after egg-laying atrophy, the expression of VG significantly decreased and the yolk gradually degraded and disappeared. LSDP plays a role in lipid accumulation and metabolism during ovarian development [43] and is coexpressed with VG, promoting ovarian maturation and yolk accumulation. In addition, the expression of SPI is significantly reduced when the ovary is redeveloping during the recovery period due to SPI or the inhibition of protein hydrolysis or proteasome production [44]. Further research is needed to determine whether a decrease in this protein reflects the process of ovarian recovery and redevelopment.

### 4.3. Food Fortification, Secondary Embryo Holding, and the Quality of Embryos and Offspring

In this study, we found that group ④ had a higher proportion of egg-holding shrimp and a higher maturity coefficient for unripe shrimp. Studies have shown that satisfying a certain protein or amino acid supply in the bait is of great significance for ensuring the normal reproduction of the parent [26]. In addition, adding appropriate animal feed to the bait could help in the development of the gonads and increase the egg-holding rate and quantity [23, 24], which was confirmed in this experiment. In addition, we found that group ④ had high SOD content and low MDA content. Studies have shown that antioxidant substances such as SOD and MDA are important aspects of the nonspecific immunity of crustaceans and could reflect the health status of the body to a certain extent. For example, a decrease in SOD and an increase in MDA are not conducive to maintaining the dynamic equilibrium of free radicals in the body and have negative effects on normal physiological metabolism [45, 46]. Therefore, the embryos in group ④ had a stronger antioxidant capacity. Combining the results of group ④ had higher hatching and survival rates, and high survival and weight gain rates of offspring. We believe that this was due to the empty-laying crayfish having undergone one egg-laying process, resulting in ovarian degeneration and atrophy, and a decrease in the embryo hatching and offspring quality of this batch of egg-holding shrimp. This was consistent with the conclusion drawn by Zhang et al. [6]. Therefore, supplementation with appropriate nutrients is necessary to promote ovarian redevelopment.

## 5. Conclusion

In this study, we observed that, on a histology level, the proportion of egg bearing shrimp in groups ③ and ④ and the proportion of stage B ovaries in group ④ were highest. The maturation coefficient of unripe shrimp of group ④ was highest. As for embracing, hatching and antioxidant index of egg effect, the egg-holding rates in groups ③ and ④ were significantly higher than those in group ①, and the proportion of bad eggs in group ④ was significantly lower than that in group ①. The hatching and survival rates of yellow eggs in group ④ were significantly higher than those in group ②. The hatching and survival rates of red eggs in groups ③ and ④ were significantly higher than those in group ①, and the survival rate of red eggs in group ④ was the highest. In terms of survival and growth rates of offspring, the survival rate of juveniles in group ① was low, and the weight gain rate of juveniles in group ④ was significantly higher than that of group ①. Analysis of related genes and proteins revealed that genes such as CTSL, VGR, and VG and proteins such as VG, LSDP, and SPI might be closely related to ovarian development. Therefore, supplementation with appropriate nutrients is necessary to promote ovarian redevelopment.

## Figures and Tables

**Table 1:** Ovarian development stage indicators of *C. quadricarinatus*.

Group	Development stage (%)	Maturity stage (%)	Recovery stage (%)
①	41.11 ± 8.39^a^	46.67 ± 5.77^a^	12.22 ± 10.72^a^
②	35.12 ± 9.16^ab^	30.36 ± 6.44^ab^	34.52 ± 5.15^b^
③	24.52 ± 4.31^b^	24.52 ± 4.31^b^	50.95 ± 8.61^c^
④	14.48 ± 2.09^c^	33.13 ± 4.47^ab^	52.38 ± 4.12^c^

All data are presented as mean ± SD. Different superscript letters indicate significant differences (*P* < 0.05).

**Table 2:** Female crayfish index of *C. quadricarinatus*.

Group	Body weight growth rate (%)	Hepatopancreas somatic indices (%)	Maturation coefficient (%)	Maturation coefficient of unripe shrimp (%)
①	−0.08 ± 5.22^a^	3.56 ± 0.99^a^	1.64 ± 1.35^a^	1.32 ± 1.09^a^
②	9.55 ± 3.90^b^	5.42 ± 0.56^a^	1.21 ± 0.64^ab^	1.23 ± 0.75^a^
③	10.30 ± 3.42^b^	5.49 ± 1.25^a^	0.83 ± 0.35^b^	1.18 ± 0.23^a^
④	9.95 ± 3.74^b^	4.71 ± 0.94^a^	1.28 ± 0.86^ab^	2.54 ± 0.72^b^

All data are presented as mean ± SD. Different superscript letters indicate significant differences (*P* < 0.05).

**Table 3:** Egg-holding index of female *C. quadricarinatus*.

Group	Egg-holding rate (%)	Absolute fecundity (egg/tail)	Relative fecundity (egg/g)	Yellow egg ratio (%)	Red egg ratio (%)	Bad egg ratio (%)
①	12.22 ± 10.72^a^	400.50 ± 16.26^a^	6.31 ± 0.38^a^	0	100	5.11 ± 0.32^a^
②	34.52 ± 5.15^b^	432.88 ± 44.61^a^	7.23 ± 1.09^a^	50	50	4.34 ± 1.35^ab^
③	50.95 ± 8.61^c^	441.90 ± 51.43^a^	7.05 ± 1.09^a^	60	40	3.05 ± 1.06^b^
④	52.38 ± 4.12^c^	439.73 ± 37.50^a^	7.34 ± 1.89^a^	54.55	45.45	1.95 ± 0.73^c^

All data are presented as mean ± SD. Different superscript letters indicate significant differences (*P* < 0.05).

**Table 4:** The hatching and survival rates of eggs incubated *in vitro* of *C. quadricarinatus*.

Group	Hatching rate of yellow egg (%)	Hatching rate of red egg (%)	Survival rate of yellow egg (%)	Survival rate of red egg (%)
①	—	80.20 ± 3.63^a^	—	50.25 ± 3.08^a^
②	69.12 ± 3.29^a^	85.09 ± 4.43^ab^	42.24 ± 5.53^a^	55.68 ± 4.08^ab^
③	72.49 ± 3.62^ab^	87.38 ± 2.59^b^	43.94 ± 6.03^ab^	59.21 ± 2.49^b^
④	76.40 ± 2.95^b^	90.67 ± 3.58^b^	49.51 ± 3.05^b^	63.53 ± 2.87^c^

All data are presented as mean ± SD. Different superscript letters indicate significant differences (*P* < 0.05).

**Table 5:** Antioxidant index of *C. quadricarinatus* egg.

Group	SOD (U/g)	MDA (nmol/mgprot)
①	334.62 ± 17.80^a^	6.50 ± 0.78^a^
②	328.04 ± 26.30^a^	6.14 ± 0.27^a^
③	375.24 ± 22.07^ab^	5.63 ± 1.08^ab^
④	421.97 ± 29.04^b^	4.19 ± 0.87^b^

All data are presented as mean ± SD. Different superscript letters indicate significant differences (*P* < 0.05).

**Table 6:** The survival rate and growth rate of offspring of *C. quadricarinatus*.

Group	Survival rate (%)	Weight gain rate (%)	Specific growth rate (%)
①	18.67 ± 2.52^a^	557.41 ± 57.82^a^	6.27 ± 0.29^a^
②	28.33 ± 3.51^b^	548.15 ± 69.91^a^	6.22 ± 0.36^a^
③	29.67 ± 3.21^b^	612.96 ± 89.29^ab^	6.53 ± 0.43^a^
④	31.33 ± 4.04^b^	640.74 ± 97.55^b^	6.66 ± 0.45^a^

All data are presented as mean ± SD. Different superscript letters indicate significant differences (*P* < 0.05).

## Data Availability

The data and material were available.

## Additional Points

*Code Availability*. Software application.

## References

[1] Zheng J. B., Cheng S., Jia Y. Y. (2019). Molecular identification and expression profiles of four splice variants of *Sex-lethal* gene in *Cherax quadricarinatus*. *Comparative Biochemistry and Physiology Part B: Biochemistry and Molecular Biology*.

[2] Cheng S., Jia Y.-Y., Chi M.-L., Zheng J.-B., Liu S.-L., Gu Z.-M. (2020). Culture model of *Cherax quadricarinatus*: temporary shelter in shed and pond culture. *Aquaculture*.

[3] Gu Z. M., Xu G. X., Huang X. M., Liu Q. W., Mi G. Q. (2003). Indoor artificial breeding and juvenile nursing of *Cherax quadricarinatus*. *Journal of Fisheries of China*.

[4] Chen Y. B., Li S. D. (2018). Key techniques for breeding of *Cherax quadricarinatus*. *Scientific Fish Farming*.

[5] Li F., Huang X. M., Shen Q. S., Li F., Bao C. L. (2013). Study on the effect of two ways for the synchronization of red claw rrayfish (*Cherax quadricarinatus*) spawning. *Journal of Biology*.

[6] Zhang H. Y., Xue T., Li G. X., Yu Y. Q. (2018). Artificial propagation techniques of *Cherax quadricarinatus*. *Scientific Fish Farming*.

[7] Wang J. Q., Xu K. (2002). Nutrient requirements of penaeid shrimp. *Journal of Dalian Fisheries University*.

[8] Kiris I. G. A., Eroldoğan O. T., Kir M., Kumlu M. (2004). Influence of neuropeptide Y (NPY) on food intake and growth of penaeid shrimps *Marsupenaeus japonicus* and *Penaeus semisulcatus* (Decapoda: Penaeidae). *Comparative Biochemistry and Physiology*.

[9] Alam M. S., Teshima S., Koshio S. (2005). Supplemental effects of coated methionine and/or lysine to soy protein isolate diet for juvenile kuruma shrimp, *Marsupenaeus japonicus*. *Aquaculture*.

[10] Palacios E., Ibarra A. M., Racotta I. S. (2000). Tissue bio chemical composition in relation to multiple spawning in wild and pond-reared *Penaeus vannamei* broodstock. *Aquaculture*.

[11] Harrison K. E., D’Abramo L. R., Conklin D. E., Akiyama D. M. (1997). Broodstock nutrition and maturation diets. *Crustacean Nutrition*.

[12] Dabrowski K., Ciereszko A. (2001). Ascorbic acid and reproduction in fish: endocrine regulation and gamete quality. *Aquaculture Research*.

[13] Pangantihon-Kühlmann M. P., Millamena O., Chern Y. (1998). Effect of dietary astaxanthin and vitamin A on the reproductive performance of *Penaeus monodon* broodstock. *Aquatic Living Resources*.

[14] Cortés-Jacinto E., Villarreal-Colmenares H., Civera-Cerecedo R., Naranjo-pάramo J. (2004). Effect of dietary protein level on the growth and survival of pre-adult freshwater crayfish *Cherax quadricarinatus* (von Martens) in monosex culture. *Aquaculture Research*.

[15] Harlıoğlu M. M., Farhadi A. (2017). Factors affecting the reproductive efficiency in crayfish: implications for aquaculture. *Aquaculture Research*.

[16] Li J. Y., Guo Z. L., Gan X. H., Wang Q., Zhao Y. L. (2010). Biochemical changes during vitellogenesis in the red claw crayfish, *Cherax quadricarinatus* (von Martens). *Aquaculture Research*.

[17] Chen Y. X., Jiang W. M., Yang Y. H., Chen X. L., Peng M., Chen X. H. (2011). Effect of several baits on the gonadal development in *Penaeus vannamei* Boone parent prawn. *Journal of Southern Agriculture*.

[18] Wen X., Chen L., Ai C., Zhou Z., Jiang H. (2001). Variation in lipid composition of Chinese mitten-handed crab, *Eriocheir sinensis* during ovarian maturation. *Comparative Biochemistry and Physiology Part B: Biochemistry and Molecular Biology*.

[19] Rodríguez-González H., Hernández-Llamas A., Villarreal H., Saucedo P. E., García-Ulloa M., Rodríguez-Jaramillo C. (2006). Gonadal development and biochemical composition of female crayfish Cherax quadricarinatus (Decapoda: Parastacidae) in relation to the gonadosomatic index at first maturation. *Aquaculture*.

[20] Liñán-Cabello M. A., Medina-Zendejas R., Sánchez-Barajas M., Herrera A. M. (2004). Effects of carotenoids and retinol in oocyte maturation of crayfish *Cherax quadrucarinatus*. *Aquaculture Research*.

[21] Zhang J. H., Kou X. M., Wang S. H., Bi J. H., Tang H. J., Jin Y. G. (2008). Primary study on effect of different for age prescription in red swamp crawfish growing and laying eggs. *Feed Review*.

[22] Zheng Y., Liu G. X., Yan W. H., Huang H. B., Peng G. (2018). A new type of incubator for artificial breeding of red crayfish and growth experiment of juveniles. *Journal of Aquaculture*.

[23] Zhang L. G., Zhong J. W., Liu Y. Q., Zhang Z. S., Zhu Y. A. (2014). Effects of light and feed on the sexual gland development of the parent shrimp of *Procambarus clarkii*. *Hebei Fisheries*.

[24] Gendron L., Fradette P., Godbout G. (2001). The importance of rock crab (*Cancer irroratus*) for growth, condition and ovary development of adult American lobster (*Homarus amer-icanus*). *Journal Experiment Marine Biology and Ecology*.

[25] Yu Z. J., Fang C. L., He G., Wang Q. P., Zhang Y. P. (2011). Effects of aquatic plants and snails on the gonadal development of *Procambarus clarkii*. *Jiangxi Fishery Sciences and Technology*.

[26] Du S. B., Hu C. Q., Shen Q. (2002). A review of dietary requirement of shrimp broodstock. *Journal of Tropical Oceanography*.

[27] Yao W. J., Huang X. H., Li H. (2009). Effect of various natural diets on broodstock’s gonad development of *Litopenaeus vannamei*. *Journal of Guangdong Ocean University*.

[28] Lu X., Peng D., Chen X. R., Wu F., Jiang M. (2020). Effects of dietary protein levels on growth, muscle composition, digestive enzymes activities, hemolymph biochemical indices and ovary development of pre-adult red swamp crayfish (*Procambarus clarkii*). *Aquaculture Reports*.

[29] Liu J. H., Zhou T. T., Wang C. G., Chan S., Wang W. (2020). Deciphering the molecular regulatory mechanism orchestrating ovary development of the Pacific whiteleg shrimp *Litopenaeus vannamei* through integrated transcriptomic analysis of reproduction-related organs. *Aquaculture*.

[30] Feng Q.-M., Liu M.-M., Cheng Y.-X., Wu X.-G. (2021). Comparative proteomics elucidates the dynamics of ovarian development in the Chinese mitten crab *Eriocheir sinensis*. *Comparative Biochemistry and Physiology Part D: Genomics and Proteomics*.

[31] Cheng S., Wei Y.-C., Chi M.-L. (2022). Mass artificial incubation of redclaw crayfish eggs in a recirculating mechanical pulling device. *Aquaculture Research*.

[32] Wu D. L., Zuo D., Huang Y. H., Ma C. A., Zhao Y. L. (2014). Cloning and expression of cathepsin L in *Cherar quadricarinatus* and its regulation by dietary vitamin C. *Journal of Fisheries of China*.

[33] Wang Z. L., Jiang J. C., Lu Y. S. (2013). Cloning and expression analysis of the Chinese L gene from pearl oyster *Pinctada fucata*. *Oceanologia Et Limnologia Sinica*.

[34] Zhao W., Chen L., Zhang F., Wu P., Li E., Qin J. (2013). Molecular characterization of cathepsin L cDNA and its expression during oogenesis and embryogenesis in the oriental river prawn *Macrobrachium nipponense* (Palaemonidae). *Genetics and Molecular Research*.

[35] Ruan Y., Wong N.-K., Zhang X. (2020). Vitellogenin Receptor (VgR) mediates oocyte maturation and ovarian development in the pacific white shrimp (*Litopenaeus vannamei*). *Frontiers in Physiology*.

[36] Wu X.-G., Yao J.-J., Yang X.-Z., Cheng Y.-C., Wang C.-L. (2007). A study on the ovarian development of *Portunus trituberculatus* in East China Sea during the first reproductive cycle. *Acta Oceanologica Sinica*.

[37] Harrison K. E. (1990). The role of nutrition in maturation, reproduction and embryonic development of decapod crustacean: a review. *Journal of Shellfish Research*.

[38] Tsutsui N., Kawazoe I., Ohira T. (2000). Molecular characterization of a cDNA encoding vitellogenin and its expression in the hepatopancreas and ovary during vitellogenesis in the kuruma prawn, Penaeus japonicus. *Zoological Science*.

[39] Jayasankar V., Tsutsui N., Jasmani S. (2002). Dynamics of vitellogenin mRNA expression and changes in hemolymph vitellogenin levels during ovarian maturation in the giant freshwater prawn Macrobrachium rosenbergii. *Journal of Experimental Zoology*.

[40] Serrano-pinto V., Landaisi I., Ogliastro M. H. (2004). Vitellogenin mRNA express in Cherax quadricarinatus during secondary vitellogenin at first maturation females. *Molecular Reproduction and Development*.

[41] Okumura T., Yamano K., Sakiyama K. (2007). Vitellogenin gene expression and hemolymph vitellogenin during vitellogenesis, final maturation, and oviposition in female kuruma prawn, *Marsupenaeus japonicus*. *Comparative Biochemistry and Physiology Part A: Molecular & integrative Physiology*.

[42] Tian H. F., Meng Y., Xiao H. B. (2014). Advances in vitellogenin research of aquatic animals. *South China Fisheries Science*.

[43] Estela L. A., Laticia R., Masakazu H. (2008). Function and structure of lipid storage droplet protein 1 studied in lipoprotein complexes. *Archives of Biochemistry and Biophysics*.

[44] Hao Z. Y., Zeng R., Shen P. H., Wu B., Jiang C. J. (2011). Progress on the function and structure of serine protease inhibitor. *Genomics and Applied Biology*.

[45] Chen Y. F. (2008). *Physiologial-Biochemistrial Effects of Scylla paramamosain to Cu^2+^ or Zn^2+^ or Interaction of Cu^2+^ and Zn^2+^ Stress*.

[46] Meng F. L., Zhang Y. Z., Kong J., Ma G. R. (1999). The research review of prophenoloxidase activating system in Crustacean. *Oceanologia Et Limnologia Sinica*.

